# Intervention of machine learning in bladder cancer research using multi-omics datasets: systematic review on biomarker identification

**DOI:** 10.1007/s12672-025-02734-6

**Published:** 2025-06-05

**Authors:** Blessy Kiruba, P. S. Athul Narayan, Badhari Raj, S. Rohieth Raj, Sam George Mathew, Sudhakaran Sajitha Lulu, Vino Sundararajan

**Affiliations:** 1 Integrated Multiomics Laboratory, School of Bio Sciences and Technology, Vellore Institute of Technology, Vellore, Tamil Nadu 632014 India; 2 Integrated Multiomics Laboratory, School of Bio Sciences and Technology, Vellore Institute of Technology, Vellore, Tamil Nadu 632014 India

## Abstract

Bladder cancer (BC) is one of the most prevalent types of cancer in developed countries. BC is characterized by its highly heterogeneous and dynamic nature, with significantly higher morbidity and mortality rates in men compared to women. Diagnosing BC requires traditional methods, such as cystoscopy, which can be invasive and costly. Recent research has heavily focused on multi-omics analysis, including genomics, epigenomics, transcriptomics, proteomics, and metabolomics, for biomarker identification. However, challenges such as computational complexity and data integration prevent these methods from achieving robust diagnostic capabilities. Hence, machine learning (ML), with its ability to process high-dimensional data and identify complex patterns, offers a promising patient outcome. By exploiting genomics, epigenomics, transcriptomics, proteomics, and metabolomics data, these models facilitate the discovery of reliable biomarkers, which are critical for early detection, prognosis, and risk stratification of the disease. Integrated models combining computational techniques with large multi-omics datasets have gained significant attention, enabling the identification of significant BC biomarkers that include genes coding for diverse cellular functions, differentially expressed genes, proteins, and metabolites. A substantial amount of multi-omics data collected from clinics and laboratories are utilized to train powerful ML models such as Support Vector Machines (SVM), random forests (RF), decision trees (DT), and gradient boosting methods (e.g., XGBoost) to perform complex tasks, including biomarker discovery, classification of subtypes and feature selection. This comprehensive review highlights the essence of integrated multiomics-ML approaches for the improvement of prognosis and diagnosis of BC.

## Introduction

Bladder cancer (BC) is the tenth most prevalent malignancy worldwide with a significant rate of morbidity and mortality [1]. Moreover, it is the fourth most prevalent cancer among men and the ninth most prevalent among women in Western countries [2]. The American Cancer Society predicts that in 2025 there will be approximately 84,870 new BC cases and 17,420 related deaths in the US [3]. Urothelial carcinoma is the most common subtype, further classified into muscle-invasive (MIBC) and non-muscle-invasive (NMIBC) forms based on the tumor penetration [4].

Despite advances in treatment approaches, including surgery, cisplatin-based chemotherapy, targeted therapy, and immunotherapy, the recurrence rate and early and accurate diagnosis remain a significant challenge [5]. The gold standard diagnostic tool, cystoscopy, is invasive, costly, and lacks sensitivity in detecting low-grade tumors, likewise, urinary cytology is limited by its low sensitivity for low-grade tumors and is prone to high rates of false positives. This highlights a critical need for non-invasive and cost-effective biomarkers with high sensitivity and specificity to improve early detection and disease monitoring [6]. Biomarkers are crucial for diagnosis, prognosis, cancer subtype differentiation, and recurrence risk assessment [7, 8]. In recent years, multi-omics exploration encompassing genomics, epigenomics, transcriptomics, proteomics, and metabolomics has gained significant attention in biomarker discovery. However, the complexity and heterogeneity of these large datasets often limit traditional statistical methods. Machine learning (ML) offers a promising alternative by applying mathematical models to learn from datasets, effectively identifying diagnostic, prognostic, and predictive biomarkers. Moreover, its application in healthcare offers benefits such as improved accuracy, early detection, efficiency, personalized medicine, and cost-effectiveness [9].

This review provides a comprehensive overview of the application of ML in BC biomarker discovery across multi-omics datasets, highlighting potential diagnostic, prognostic, and therapeutic advancements for improved patient outcomes.

## Machine learning in multi-omics studies

### Benefits of machine learning in multi-omics

The Tumor Microenvironment (TME) is extremely complex and heterogeneous, comprising various cell types, including tumor cells, immune cells, blood vessels, extracellular matrix, and fibroblasts. This complex network can make it difficult to identify biomarkers [10]. Therefore, ML could be used to identify potential biomarkers from the immense amount of multi-omics data associated with TME. Cost-effectiveness is another aspect, where a study depicted an average cystoscopy to be around $430 on average for patients in the US [11]. Additionally, bladder cancer also requires lifetime cystoscopic surveillance, making it the most expensive cancer to treat per patient [12]. However, ML models can lower unnecessary cystoscopies by 30%− 40% in NMIBC patients [13]. ML is particularly crucial in biomarker discovery due to the complexities of large, high-dimensional datasets. Traditional statistical methods like t-tests and ANOVA are limited because they assume specific data distributions (e.g., normality), often violated in genomic data. Moreover, deep sampling can create non-linear relationships, outliers, and distributions with kurtosis, complicating analysis. As dataset complexity grows, conventional methods become computationally infeasible [14].

There are various ML algorithms used in multi-omics data analysis. For instance, algorithms like Boruta, SVM-RFE, and pamr, are used to detect the most relevant key genes, proteins, or metabolites that can be utilized as biomarkers for prognosis or diagnosis and to differentiate between cancer subtypes [15]. By focusing on key features, these algorithms reduce computational burden while enhancing efficiency. Moreover, Classification algorithms, such as Random Forest, XGBoost, SVM, KNN, and decision trees, are used to classify patients into risk groups based on their molecular profiles, enabling personalized treatment decisions. Additionally, Regression methods, such as LASSO, Ridge, and Enet, are employed to identify biomarkers linearly associated with disease severity or treatment response, reducing dimensionality in omics datasets while preserving predictive power. Lastly, survival analysis algorithms like coxBoost, Stepwise COX, and plsRcox model the relationship between dataset parameters and survival time, allowing for the identification of biomarkers that predict patient prognosis. These survival analysis techniques are particularly valuable for uncovering biomarkers with complex, non-linear associations with outcomes, which might be overlooked by other methods [16].

Furthermore, the algorithm selection largely relies on the type of data being analyzed. For instance, LASSO and SVM are particularly effective for high-dimensional datasets, where feature selection and classification are critical. In contrast, Random Forest is better suited for handling relatively less complex datasets. Therefore, selecting the appropriate ML algorithm, applying effective feature selection methods, optimizing model parameters, and evaluating model performance are critical for ensuring robust predictions and meaningful biological interpretations. Following the selection of an algorithm, various systematic approaches are employed to optimize its hyperparameters, which are manually assigned before training and govern the model's learning process. This is particularly important to prevent overfitting and ensure consistent performance. The choice of hyperparameter optimization techniques depends on the user’s preference and computational constraints. A more efficient approach involves Bayesian optimization, a probability-based technique, or Grid Search, which systematically assesses all possible combinations of hyperparameters to determine the most effective setting. During training, the algorithm learns its parameters, such as coefficients and intercepts in the case of a linear regression model, based on the provided data to improve the model’s predictive performance [17].

**Fig. 1:**
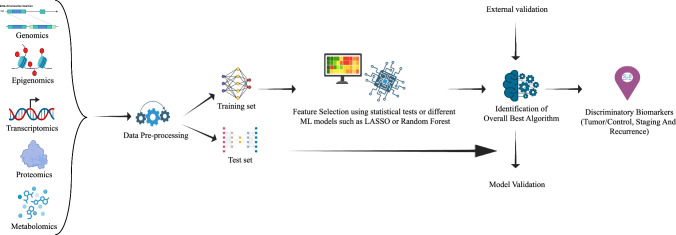
Schematic representation of the workflow for biomarker identification using machine learning. Multi-omics data, including genomics, epigenomics, transcriptomics, proteomics, and metabolomics, are collected from patient samples and pre-processed (e.g., normalization, batch correction). The data is divided into training and test sets, with the training set undergoing feature selection to identify key genes or molecules using statistical tests or machine learning models. An appropriate algorithm is selected for further filtering, and external datasets are used for validation. The test set is employed to evaluate the model using metrics such as accuracy, ROC-AUC, sensitivity, and specificity. Biomarkers are ultimately selected based on their contribution to model performance and biological relevance, providing insights into potential diagnostic, prognostic, or therapeutic applications [18]

A schematic workflow representing the identification of biomarkers by ML algorithms using omics datasets is given in Fig. 1.

### Common machine learning algorithms

LASSO is a linear regression model derived from traditional ordinary linear sqaure (OLS) regression. Its objective is to minimize the cost of the regression model while improving feature selection. LASSO achieves this by including a penalty term to OLS model. As a result, the model automatically shrinks the coefficients of less important features to zero, effectively eliminating trivial features [19]. For a given set of observed values *X*_*i*,_ where *i* = *1, 2, ….n*, the LASSO cost function is defined as:

$$ \mathop {\min }\limits_{\beta } \left( {\Sigma \left( {y_{i} - X_{i} \beta } \right)^{2} + \lambda \Sigma \left| {\beta_{j} } \right|} \right) $$

The objective is to determine the optimal regression coefficients β\betaβ that minimize the total cost function. The L1 regularization term |*β*_*j*_| induces sparsity by forcing some coefficients to zero. Upon solving for *β*, the resultant coefficient vector *β* = (*β1, β2,…,* β_j_) is obtained. The penalty term is strictly controlled by a regularization parameter λ that determines the degree of shrinkage applied to the coefficients. Ridge regression, in contrast, applies the L2 regularization penalty that penalizes large coefficient magnitudes rather than forcing them to zero [20]. Elastic Net (Enet) Regression combines both L1 (LASSO) and L2 (Ridge) penalties. In biomarker discovery using large and high-dimensional datasets, LASSO is applied to eliminate less important features, improving its performance while reducing noise that might overfit the data [21].

Support Vector Machine (SVM) is widely used for both regression and classification analysis. SVM finds and optimizes a hyperplane that acts as a (*d –1*)-dimensional classification boundary in a *d*-dimensional dataset, ensuring better generalization [22]. Given a dataset with *n* data points:

$$D = \{\left({x}_{i}, {y}_{i}\right){\}}_{\left\{i=1\right\}}^{\left\{n\right\}}, {x}_{i}\in {\left\{R\right\}}^{d}, {y}_{i}\in \{-1, 1\}$$

where:

- *x*_*i*_​ is the feature vectors in a *d*-dimensional space
- *y*_*i*_​ is the class label (+ 1 or -1).

A hyperplane in *d*-dimensional space is given by:

$${w}^{T} x + b = 0$$

where:

- *w* is the weight vector (normal to the hyperplane).
- *b* is the bias term

Classification is perfect when:

$${y}_{i}\left({w}^{T}{x}_{i}+ b\right)\ge 1, \forall i$$

SVM can be solved using kernel function (kernel-SVM) or Lagrangian Dual Formulation to handle complex datasets. SVM is a widely used classification algorithm in bioinformatics recognized for its robustness and handling of complex biological data [23]. Similarly, Decision Trees (DT) have applications like those of SVM. DT is a recursive algorithm that splits features based on a threshold value, forming a hierarchical tree-like structure, using criteria such as Gini Index or Shannon Entropy [24]. However, DTs are prone to overfitting, which can be mitigated by Random Forest (RF), an ensemble learning method that constructs multiple decision trees using bootstrap aggregation (bagging). It follows a Bootstrap Aggregation approach to aggregate their outputs. RF improves classification performance and robustness, making it highly effective in analyzing multi-omics datasets [25]. K-Nearest Neighbors (K-NN) is is another widely used algorithm for both regression and classification tasks, which determines the class or value of an input point by analyzing its *k-nearest neighbors*. The closeness of data points is generally measured using the Euclidean distance or other distance metrics, such as the Manhattan distance. The class label is assigned centered around the majority vote among the *k-nearest neighbors* [26]*.* K-NN is highly flexible since it does not assume any prior distribution about the data. The choice of *k* determines the performance of the model. A small *k* might cause overfitting due to sensitivity to noise, whereas a large *k* may lead to underfitting. K-NN struggles with high-dimensional data. Therefore, it is essential to apply dimensionality reduction techniques, such as Principal Component Analysis (PCA), to improve its performance and efficiency [27, 28].

Furthermore, the performance of the various ML models is evaluated with evaluation metrics. The model evaluation decides whether a model will generalize to unseen data and biases through various techniques and metrics according to the type of model. For classification algorithms, the confusion matrix divides the test samples based on the true and predicted values: true positive (TP), correctly predicts a positive instances, true negative (TN) correctly predicts a negative instances, a false positive (FP) is incorrectly predicting a positive instances, and a false negative (FN) is incorrectly predicting a negative instances. One of the basic metrics derived from these values is accuracy, which measures the proportion of correct predictions:

$$Accuracy=\frac{TP+TN}{TP+TN+FP+FN}$$

However, accuracy can be misleading in imbalanced datasets. In such cases, precision recall (sensitivity) and specificity offer more informative assessments. The formulas are given as:

$$Precision=\frac{TP}{TP+FP}$$

$$Specificity=\frac{TN}{TN+FP}$$

$$Sensitivity/Recall=\frac{TP}{TP+FN}$$

Importantly in diagnostics, sensitivity/recall are considered as true positive, precision as positive predictive label, and specificity as true negative. Furthermore, the F1 score is the harmonic mean of precision and recall that provides a balanced evaluation:

$$F1 Score=\frac{2*Precision*Recall}{Precision+Recall}$$

Another robust measure is the Mathhew’s correlation coefficient (MCC), which accounts for the correlation between the real and predicted values of the given instances.

$$MCC=\frac{TP*TN-FP*FN}{\sqrt{\left(TP+FP\right)*\left(TP+FN\right)*\left(TN+FP\right)*(TN+FN)}}$$

Additionally, the AUC-ROC curve evaluates a model’s ability to distinguish between classes by plotting the true positive rate against the false positive rate across different thresholds. A higher area under the curve (AUC) indicates better classification performance.

For the regression algorithms, i.e., tasks like predicting a continuous variable based on one or more other variables, the evaluation measures how closely the model predicts the value of the dependent variable based on an independent variable to the actual value. One of the most common metrics include the Pearson’s correlation coefficient, which is defined as:

$$r=\frac{{\sum }_{i=1}^{n}\left({x}_{i}-\overline{x }\right)\left({y}_{i}-\overline{y }\right)}{\sqrt{{\sum }_{i=1}^{n}{\left({x}_{i}-\overline{x }\right)}^{2}{\sum }_{i=1}^{n}{\left({y}_{i}-\overline{y }\right)}^{2}}}$$

Another key metric, R^2^ (coefficient of determination), represents the proportion of variance in the target variable *y* that is accounted for by the predicted values $\widehat{y}$, in comparison to the variance explained by the mean $\overline{y }$​ in the test dataset. An R^2^ = 1 indicates a perfect fit, while R^2^ = 0 suggests no useful prediction. It is defined as:

$${R}^{2}=1- \frac{SS(y-\widehat{y})}{SS(y-\overline{y } )}$$

where SS denotes the sum of squares calculated on the test data. Moreover, in regression models, error calculations play a crucial role in quantifying how close the predicted values are to the actual values. Mean Absolute Error (MAE) is a simple metric that computes the average absolute difference between the predicted and actual values, indicating overall error. Additionally, Mean Squared Error (MSE) and Root Mean Squared Error (RMSE) take these differences a little further by squaring them, which penalizes large errors more compared to small ones. Further, these give the error in the outcome scale. An extended version of MSE is the Mean Absolute Percentage Error (MAPE), which is also a popular metric that presents the error in percentage terms, giving a more interpretable view of the respective error in terms of scales of actual values [29, 30].

## Multi-omics data in bladder cancer

### Genomics

Genomics represents the complete DNA sequence of an organism and plays a critical role in identifying cancer-specific biomarkers as a basis for diagnosis, prognosis, and personalized therapy [31]. It is identified as the most relevant field in the research of cancer as the disease progresses due to changes or variations the genetic material. The genomic alterations associated with cancer, includes SNPs, mutations in coding regions, genomic loss or amplification, chromosomal rearrangements, aberrant methylation, and changes in gene expression [32–34]. Machine learning algorithms, with the help of bioinformatics tools are used to identify potential bladder cancer biomarkers, where it was trained on gene expression data to distinguish between the phenotypes of bladder cancer [35, 36].

A study conducted by Liosis et al. used the elastic-net regularized logistic regression, which combines LASSO and ridge regression to handle high-dimensional genomic data. With statistical modelling, it led to the identification of two novel gene signatures for bladder cancer, where 55 genes including ABCC2, OASL, PROM2 are correlated with Neoadjuvant chemotherapy therapy response, achieving an accuracy rate of 77.42% with an AUC of 0.7174, alongside 57 genes including ACBD7, DAPK2, CLDN6 are associated with BCa disease progression, with an impressive accuracy of 92.11% of AUC 1.0 [37]. This capability to identify specific genetic markers was crucial for tailoring personalized treatment strategies, which allowed clinicians to predict patient outcomes more effectively. Such advancements in biomarker discovery are vital, especially in a landscape where individualized therapy is becoming the norm in cancer management.

Similarly, Barbour et al., evaluated the association of ERCC2 mutations with bladder cancer prognosis through whole-genome sequencing data. They found that ERCC2 mutations were independently associated with favourable prognosis in bladder cancer and it was a helpful clinical marker for risk stratification and treatment decisions. To further elucidate the clinical relevance of ERCC2 mutations, a SVM model was developed by exploiting the altered patterns of somatic mutation distributions in tumours harbouring ERCC2 mutations. The SVM model was able to predict, with high accuracy, whether a given ERCC2 mutation was pathogenic or not and this would be useful to identify patients likely to respond to cisplatin therapy [38]. The clinical significance was further confirmed by another related study where the researchers analysed pretreatment transurethral resection material from patients with MIBC who received NAC before radical surgery. On analysis, they found out that deleterious mutations in ERCC2 strongly correlated with pathological response to NAC. Interestingly, ERCC2 mutations accounted for 13% in responders but only 2% in non-responders. This indicates that ERCC2 mutation status could be a very important biomarker for predicting the efficacy of NAC in bladder cancer patients and thus could direct the decision-making process regarding treatment, which may benefit the patient [39].

Furthermore, the study done by Yu et al. employed the use of the GenEpi ML model where it could identify interactions between genetic variants like within-gene and cross-gene interactions linked to bladder cancer phenotypes [40]. The study recruited Caucasian participants who are controls in the UK Biobank, matching cases to controls with age, sex, BMI, and smoking status. Four interactions including ST7L-ADSS2, FHIT-CHDH, LARP4B-LHPP, and RBFOX3-MPRIP were replicated in an independent set of 856 non-Caucasian participants who were associated with bladder cancer risk, where they developed an iPRS (Interaction-empowered polygenic risk score) that incorporates the interactions which showed a hazard ratio of 1.81 when comparing the highest tertile to the lowest tertile, and achieved an AUC of 0.91 which indicated an enhanced bladder cancer screening [41].

Additionally, Li et al. developed a novel diagnostic model to identify biomarkers in mitochondria-related genes as its dysfunction played a role in bladder cancer progression. LASSO and SVM-RFE algorithms were used and major biomarkers like GLRX2, NMT1, OXSM, TRAF3IP3 were identified, which had an AUC of 0.912 which meant mitochondria-related genes help in early detection of bladder cancer which may aid in patients' identification who would benefit from aggressive monitoring or intervention [42]. Similarly, Qian Yu et al., resorted to computational methods for studying the expression of genes involved in immuno-regulation. Using such study, the NR4A1 gene is shown to be an essential prognostic biomarker, which impacts on bladder cancer invasion through the involvement of immune cells like regulatory T cells and macrophages [43]. It establishes the incorporation of these immune cell interactions into bladder cancer, which would eventually characterize the disease progression or response to treatments.

Despite the advancement in machine learning for the exploration of biomarkers for bladder cancer, certain drawbacks exist. The biomarkers that are discovered using machine learning models should be verified in the laboratory. Moreover, there is a challenge posed by the limited availability of large well-annotated genomic databases for bladder cancer that can impede the construction of effective ML models. The genomic profiling of patients suffering from bladder cancer with African American, Asian American, and Tunisian origins revealed that AKT levels were substantially higher in these groups as compared to European American patients featured above underscored the importance of considering diverse populations in the study of genomes to identify the biomarkers that may hold relevance to clinical practice [44]. Table 1 presents key biomarkers identified from genomic data using various machine learning approaches, detailing their functions, associated pathways, regulators, and their roles as biomarkers in other cancers based on the GeneCards database and literature.

**Table 1:** Prominent biomarkers found by ML models using Genomic data

Biomaker	Function	Associated pathways*	Regulating transcription factors*	Reported in other cancers	Biomarker type	References
ABCC2	Efflux transporter by actively pumping chemotherapeutic drugs out of cancer cells	Drug ADME and Statin Pathway	FOXC, FOXD3 FOXI1, FOXJ2, FOXL1, GATA-2 GATA-3, HFH-3, Sox5	Gastric	Prognostic	[37, 45, 46]
OASL	A crucial prognostic and immunological biomarker and play an important role in the tumour microenvironment	Antiviral mechanism by IFN-stimulated genes	Elk-1, Ik-2, IRF-2, NRF-2, Olf-1, PPARgamma1/2, RFX1, STAT1alpha/beta	Breast	Prognostic	[37, 47, 48]
PROM2	Its upregulation inhibits ferroptosis through facilitated iron export and promotes tumorigenesis	Ferrintin pathway	c-Myc, CP2, GATA-6, Max, MyoD, NF-1, NF-1/L, p53	Lung squamous cell carcinoma	Prognostic and Diagnostic	[37, 49, 50]
ACBD7	Energy regulation via leptin-melanocortin pathway and involved in cell proliferation and differentiation	Insulin-like growth factor signaling pathway	C/EBPbeta, CUTL1, Evi-1, LyF-1, NF-AT, NF-AT1, NF-AT2, NF-AT3, NF-AT4, NRF-2	Thyroid	Diagnostic	[37, 51–53]
DAPK2	Upregulation causes the transition of autophagy to apoptosis	AKT1/CyclinD1 pathway	AhR, Arnt, C/EBPalpha, CREB, MyoD, Pax-4a, POU2F1, POU2F1a, Roaz, ZID	Colorectal	Prognostic	[37, 54]
CLDN6	A tight junction protein that increases drug resistance by altering cell adhesion	PI3K/Akt signaling pathway	AP-1, ATF-2, c-Jun, COUP, COUP-TF	Ovarian Prognostic		[37, 55, 56]
GLRX2	Involved in protein folding, redox regulation, and is associated with mitochondrial biological activity that regulates its function and energy production processes such as ATP synthesis	PAK Pathway	aMEF-2, E47, GATA-1, ITF-2, MEF-2A	Oral squamous cell carcinoma	Prognostic	[42, 57]
NMT1	Involved in protein modification and signalling processes and promotes tumour	Apoptotic pathway and Programmed Cell Death	AP-1, c-Fos, c-Jun, Cart1, CBF (2), CBF-A, CBF-B, CBF-C, IRF-1, MAZR	Breast cancer	Prognostic	[42, 58]
TRAF3IP3	Codes proteins that play an important role in various cellular functions	c-Jun N-terminal kinase signal transduction pathway	AML1a, AP-1, ATF-2, c-Jun, NF-kappaB, NF-kappaB1	Breast	Prognostic	[42, 59]
ERCC2	Encodes a DNA helicase essential for nucleotide excision repair, a process critical for correcting DNA damage. Its mutations were an independent predictor for prognosis in bladder cancer patients	RNA Polymerase II Transcription Initiation and Promoter Clearance	AML1a, CBF(2), En-1, GR, Ik-2, LCR-F1, MZF-1, p53, ZID	Breast	Prognostic	[38, 60]
ST7L-ADSS2	ST7L is part of the Wnt/GSK-3β/β-catenin signalling pathway. Contact of this ADSS2 with ST7L might inhibit the tumour suppressive role of the latter for the development of BC	Denovo pathway and in the salvage pathway of purine nucleotide biosynthesis	CUTL1, FOXC1, IRF-7A, NRSF form 1/2, Pax-5, Roaz, STAT3, USF1, USF2	N/A	Diagnostic	[41]
FHIT-CHDH	FHIT is a known tumour suppressor, and aberrant expression in this gene leads to decreased apoptosis and increased resistance to genotoxic agents. The association of CHDH with arsenic metabolism may relate to arsenic exposure, an established risk factor for BC	Methionine de novo and salvage pathway, One-carbon metabolism and related pathways	c-Ets-1, GATA-2, ITF-2, NF-kB, Tal1B, YY1	N/A	Diagnostic	[41]
LARP4B-LHPP	Their interactions inhibit the proliferation of BC cells by inhibition of the AKT/p65 pathway	Pyrophosphate hydrolysis pathway	AP-1, AREB6, ARP-1, GCNF, ISGF-3, Pax-2	N/A	Diagnostic	[41]

^*^Taken from Genecards database [61]

*N/A* not available

### Epigenomics

Genes that undergo epigenetic modification can lead to uncontrolled cell growth and division; in the case of the urothelial lining of the bladder, this may lead to urothelial cancer (UCa). Several epigenetic modifications such as methylation, acetylation, phosphorylation, SUMOylation have been identified in the progression of cancer. Considering the dynamic nature of biological systems, ML can be utilized to analyze large datasets of epigenetic data to identify biomarkers that have undergone significant epigenetic modifications. The necessity of these studies is to improve the prognostic and diagnostic significance in BCa.

Multiple independent studies utilized ML to analyze BCa prognostic markers and to develop models in order to achieve a reliable prognostic tool, also to improve BCa survillance in patients. A study conducted by Zhang et al. [62] employed 101 combinations of 10 different algorithms to identify genes that exhibit aberrant methylation, which may serve as potential epigenetic biomarkers. From this analysis, nine genes were identified based on the C-indices (concordance indices), with StepCox and survivalSVM recognized as the top-performing algorithms. Following this, the identified genes underwent further evaluation of their prognostic capabilities. Ultimately, three genes-RNH1, TAP1, and AHNAK were selected to construct a nomogram along with age and tumor stage, which were identified as independent factors through both univariate and multivariate Cox analyses of the nine genes. Similarly, Jiang et al. [63] developed a machine learning-based urinary DNA methylation diagnostic panel to distinguish BCa patients from non-BCa patients by using genes that have undergone aberrant methylation. The performance of the panel was evaluated, and three individual genes with significant prognostic potential were identified: ZNF671, OTX1, and IRF8, which achieved an impressive area under the curve (AUC) values of 0.86, 0.82, and 0.81, respectively, indicating effective classification performance. In contrast, Köhler et al. [64] applied three machine learning methods (random forest (RF), boosted trees (BT), and LASSO) in tandem to the urine samples, identifying 65 abnormally methylated CpG sites and arrived at a combination of biomarkers, which led to the construction of a decision matrix for an array of all possible combinations ranked for their prognostic ability. Notably, the combination of ALOX and TRPS1 manifested the highest sensitivity of 61%. In addition, van der Heijden et al. [65] developed a urinary methylation biomarker diagnostic classifier designed to enhance the monitoring of BCa and reduce the reliance on invasive cystoscopy procedures in patients undergoing surveillance for BCa. Urine samples were analyzed, and using logistic regression, a model was developed with a combination of three markers CFTR, SALL3, and TWIST1 that outperformed other combinations with a formidable AUC value of 0.87. Furthermore, the combined analysis of the patients’ cytological data and the three-gene methylation classifier was superior to the analysis of cytological data alone. Additionally, the study indicated that this combined approach could have prevented cystoscopies in 36% of patients within the validation cohort that was used to assess the model.

In addition to the identification of biomarkers, recent studies have increasingly focused on evaluating potential machine learning-based BCa risk predicting models, to substantially enhance diagnostic and prognostic capabilities across diverse medical domains. For instance, Mohanad et al. [66] analysed aberrant methylation patterns in promoter sequences of DNA damage repair (DDR) genes and found 154 DDR genes that have undergone aberrant methylation in BCa patients. Following hierarchical clustering of 154 genes, 12 genes were selected. Notably, two genes that revealed significant methylation in their promoter regions: MutS homolog (MSH4) and Retinoblastoma binding protein 8 (RBBP8), commonly referred to as C-terminal binding protein (CtBP)-interacting protein (CtIP), were culled for model development. Hypermethylation of RBBP8 alters DNA damage repair pathways [67]. The human MutS homolog 5 (hMSH5) maintains genome stability by inhibiting double-strand breaks (DSBs) through Non-homologous end joining (NHEJ) pathway. hMSH5 lead to the formation of 53BP1 foci as DSB sites. They are protective in nature and hMSH5 deficiency leads to the slow formation of 53BP1 [68]. Several predictive machine learning algorithms (Decision Tree (DT), Kernel Support Vector Machine (SVM), K-Nearest Neighbor (KNN), Logistic Regression (LR), and Random Forest (RF) were employed. Among these, the KNN algorithm achieved an outstanding accuracy of 90.05 ± 4.5%, indicating excellent predictive performance.

A notable study conducted by Chatterjee et al. [69] investigated the potential of a BCa prognostic risk model (risk score) utilizing a signature gene panel by combining methylation, RNAseq and patients’ clinical data analyses. Methylation data were collected and subjected to differential analysis to identify prognostic genes whose methylation status varies significantly. Subsequently, the most significant popyrognostic genes were subjected to network analysis, which revealed six hub genes (given in table) that were most suitable for developing the risk model. It was demonstrated that the model can independently predict the survival of BCa patients. A similar study by Peng et al. [70] utilized methylation data from The Cancer Genome Atlas Program (TCGA) to develop a multiclass prediction system and built a methylation-based model to identify significant biomarkers for predicting common urothelial carcinomas such as bladder urothelial carcinoma (BLCA), renal clear cell carcinoma (KIRC) (also known as clear cell renal cell carcinoma (ccRCC)), a variant of renal carcinoma characterized by distinct clear cell histology and prostate-adenocarcinoma (PRAD), a neoplastic lesion of the prostate gland. The multiclass prediction system distinguished between normal tissue and cancer tissue and the origin to which it belongs. Remarkably, this model was also able to differentiate between metastatic lymph nodes and normal lymph nodes. Furthermore, the study also examined the association between their methylation model and tumor node metastasis (TNM) stage. The performance of the methylation model alone was superior and the combination of the two proved to be a promising prognostic tool.

One intriguing study by Guo et al. [71] intended to develop a novel methylation risk model for bladder cancer (MRSB) by selecting various candidate CpG sites using the support vector machine-recursive feature elimination (SVM-RFE) and least absolute shrinkage and selection operation (LASSO) methods. The eight candidate genes that were identified are subsequently used to build the model, that includes APC, SLCO4A1, ZC3H3, ADARB2, COL9A2, KRTDAP, POU3F3 and TNFAIP8L3. An impressive AUC value of 0.86 was obtained for the developed MRSB, which corroborates its predictive ability. Additionally, an effective nomogram was constructed to predict the individual progression risk, integrating factors such as the methylation risk score (MRSB), sex, age and tumor stage.

The integration of machine learning and DNA methylation analysis is proving to be a transformative approach in the early detection and prognosis of bladder cancer. Studies have successfully identified multiple genes with aberrant methylation that serve as potential biomarkers, demonstrating high predictive accuracy through various models and also significantly enhancing the quality of care and treatment results for patients with bladder cancer. The prominent biomarkers found using epigenomic studies by ML models along with their functions associated pathways, regulators, and their roles as biomarkers in other cancers based on the GeneCards database and literature are given in Table 2.

**Table 2:** Prominent biomarkers found by ML models using epigenomic data

Biomaker	Function	Associated pathways*	Regulating transcription factors*	Reported in other cancers	Biomarker type	References
TAP1	Functions in antigen presentation, facilitating immune response through MHC class I molecules. Downregulation of the protein causes the cancer cells to evade immunosurveillance	Antigen processing-Cross presentation and Class I MHC mediated antigen processing and presentation	AML1a, STAT3	Ovarian	Prognostic and Diagnostic	[18, 62, 72, 73]
AHNAK	Serves as a scaffold protein, participating in cell signalling and cytoskeletal organization. The upregulation of AHNAK is observed in BC tissues	Phospholipase-C Pathway	Bach1, Egr-2, Elk-1, FOXD3, HTF, LUN-1, Pax-5, RelA, Spz1	Breast	Prognostic and Diagnostic	[62, 74–76]
RPP21	An essential subunit of the RNase P complex is involved in the maturation of tRNA by cleaving its precursor forms, thereby promoting proper protein synthesis, and also alongside RPP29, is involved in the DNA damage response	rRNA processing in the nucleus and cytosol and tRNA processing	E2F, Nkx2-5, p53, PPAR-gamma1/2	Liver	Prognostic and Diagnostic	[62, 77–79]
ZNF671	Functions as a tumor suppressor, inhibiting cell proliferation and metastasis	RNA Polymerase II Transcription pathway	AREB6	Cervical	Prognostic and Diagnostic	[63, 80, 81]
OTX1	Acts as a transcription factor that promotes oncogenesis	Beta-2 adrenergic-dependent CFTR expression pathway	AREB6, E2F, HOXA5, POU2F1, SRY	Ovarian	Prognostic and Diagnostic	[63, 82, 83]
IRF8	Involved in regulating immune responses and myeloid cell differentiation	NF-kappaB Signaling pathway, Type II interferon signaling pathway, Toll-Like Receptors Pathway	C/EBPbet, COMP1, E4BP4, Evi-1, FOXO3b,HNF-1	Rectal	Prognostic and Diagnostic	[63, 84, 85]
ALOX	Encodes arachidonate 5-lipoxygenase, crucial for leukotriene synthesis and inflammation	Biosynthesis of DPA-derived SPMs, Biosynthesis of specialized proresolving mediators (SPMs) and NF-kappaB Signaling pathways	AP-1, ATF-2, c-Jun, Egr-1, GR, p53, Sp1	Breast	To construct a decision matrix	[64, 86, 87]
TRPS1	A marker that is crucial in metastasis and identifying triple negative breast cancer	N/A	aMEF-2, E2F, HFH-1, MEF-2A, STAT3	Breast	To construct a decision matrix	[64, 88]
CFTR	Encodes the cystic fibrosis transmembrane conductance regulator, crucial for chloride ion transport in epithelial cells	AMPK Enzyme Complex Pathway	AP-1, ATF-2, c-Jun, Sp1, SRF, YY1	Colorectal	Monitoring Bladder cancer	[65, 89, 90]
SALL3	A transcription factor involved in embryonic development and tissue patterning	N/A	E2F, Evi-1, FOXL1, POU3F2	Liver	Monitoring Bladder cancer	[65, 91, 92]
TWIST1	A transcription factor that regulates transition from epithelial cells to mesenchymal cells (EMT), facilitating cell migration and invasion during development	Notch-mediated HES/HEY network pathway, Cell migration and invasion through p75NTR and CKAP4 signaling pathway	AP-1, AREB6, ATF-2, c-Jun, MyoD, p53, Pax-3, STAT3	Breast	Monitoring Bladder cancer	[65, 93, 94]
RBBP8	An endonuclease involved in DNA damage repair and cell cycle checkpoint control	G2/M DNA damage checkpoint pathway	Egr-3, FOXJ2, Nkx2-2, Nkx2-5, SRF	Gastric	Prognostic and Diagnostic	[66, 95, 96]
MSH4	Repair of DNA mismatches during homologous recombination	Cell cycle and meiotic pathway	AML1a, AP-4, C/EBPalpha, E4BP4, Evi-1, FOXO3, POU2F1	Colorectal	Prognostic and Diagnostic	[66, 97, 98]
EGFR	Encodes the epidermal growth factor receptor, which is crucial for cell growth, differentiation, and proliferation and driver of cancer progression	Cellular Apoptosis Pathway, Signaling by EGFR in Cancer, GPCR Pathway	c-Myc, Max1, p53, STAT3	Non-Small Cell Lung cancer	Prognostic	[69, 99, 100]
NFE2	A transcription factor playing an important role in erythropoiesis and antioxidant defence	RNA Polymerase II Transcription pathway, Generic Transcription Pathway	AML1a	Liver	Prognostic	[69, 101, 102]
ARL4D	Involved in cell signaling and cytoskeletal organization, it influences processes like cell migration through its effects on cytoskeletal dynamics	N/A	AML1a, ATF-2, c-Jun, FOXJ2, NF-kappaB, Nkx5-1, Pax-5, USF1	Gastric	Prognostic	[69, 103, 104]
SH3RF2	An E3 ubiquitin ligase that regulates protein degradation and cellular signaling pathways	Anti-apoptotic regulator of the JNK pathway	AML1a, Arnt, Cdc5, E47, Gfi-1, HSF2, Lmo2, POU6F1, Tal-1beta	Ovarian	Prognostic	[69, 105, 106]
CDH3	Encodes cadherin 3 (P-cadherin), which regulates aspects of cell adhesion and maintaining tissue architecture	ERK signalling pathway	AP-1, AREB6, ATF-2, c-Jun, COUP, HNF-4alpha1, Sp1	Colorectal	Prognostic	[69, 107, 108]
TNFAIP8L3	Involved in regulating inflammation and apoptosis	Enhance the activity of the PI3K-AKT and MEK-ERK pathways	AREB6, ATF6, FOXC1, HFH-3, NF-AT, TBP	Head and Neck	To build MRSB	[71, 109, 110]

^*^Taken from Genecards database [38]

*N/A* not available

### Transcriptomics

Transcriptomics is the study of the total RNA transcripts produced from a genome. It aims at analysing the gene expression, regulation, and, most importantly, cellular functioning patterns [111]. These RNA molecules can be measured and analysed using methods like RNA sequencing, which would grant scientists the knowledge about how genes get activated, interact, and respond to other biological or environmental stimuli [112]. This is critical in establishing comprehensive analysis of gene expression and regulation therapeutic targets in domains like developmental biology, personalized medicine, and disease research [113]. Transcriptomic studies represent a key method of identifying biomarkers for bladder cancer by yielding insights into the molecular mechanisms underlying the disease. From analysing the total set of RNA transcripts within cancer cells, gene expression patterns can be found to distinguish bladder cancer from healthy tissue [114]. These studies enable the discovery of potential biomarkers that can serve as indicators for cancer diagnosis, prognosis, or treatment response [115]. Following the advancement of high-throughput sequencing and bioinformatics, with transcriptomic data, classifications of bladder cancer subtypes are more precise and assist in personalized treatment strategies [116].

A study by Wang et al. analysed gene expression datasets using ML models such as LASSO logistics regression and identified the overexpressed gene PTHLH as a biomarker. Moreover, it was observed to be a prominent biomarker for BC with excellent diagnostic and prognostic capability with a satisfactory prediction efficiency of AUC = 0.775. In vitro validation done by knocking out the PTHLH gene from BC cells and using the co-culture method and immunofluorescence demonstrated supporting evidence of the involvement of PTHLH in BC proliferation [117]. In another study, the role of SMAD6 in BC proliferation was studied by Chen et al., with mRNAseq data using LASSO-Logistic Regression, Boruta, random-forest, svmREF, and XGboost ML models. In this transcriptome study, SMAD6 was found to be a evident prognostic biomarker with the downregulation of SMAD6 resulting in the inhibition of BC proliferation. Wound healing assays and colony forming assays were performed for validation, and sufficient evidence was obtained [118]. Zhang et al., conducted a transcriptome study using bulk RNAseq and single cell RNA seq data of BC to find a novel biomarker. The analysis of the data was done using CoxBoost ML model and LDLRAD3 was found as a prognostic biomarker with great predictive efficiency. The upregulation of LDLRAD3 expression was found to be a leading cause for poor prognosis of BC patients. The results were further validated using invivo mouse models [119].

Another study done by Song et al., analysed the gene expression data and single cell transcriptome data using ML models such as LASSO and Logistic regression model, and found LIG1 to be upregulated in BC tissues, leading to poor prognosis. LIG1 was also able to show good prognostic prediction of BCa with a prediction efficiency of AUC = 0.793 and identified as a prognostic biomarker of BCa [120]. Another transcriptomic study by Shen et al., analyzed gene expression and single-cell RNASeq datasets using the ML models SVM-RFE and LASSO. Further, PPP2R2B was found to be a prognostic biomarker for BC, with PPP2R2B being downregulated in BC tissues [121]. Li et al., conducted a similar transcriptomic study with single cell and bulk RNAseq data using the ML models CoxBoost, Enet, Lasso, RSF, plsRcox, GBM, Stepwise Cox, Boruta, pamr, and Ridge. After the analysis, the study generated 3 novel prognostic biomarkers FN1, CRYAB, and COL6A1 with good prediction accuracy [122]. Another transcriptomic study utilized 10 different ML models and found that SVM model was the best one among them in predicting the prognosis of BC. It also identified FOXP3 and TOX as prominant prognostic biomarkers for BC. The upregulation of FOXP3 and downregulation of TOX was found to be the leading cause for poor prognosis among BC patients [123].

By reviewing these different transcriptomic studies, SVM AND LASSO logistic regression model was found to be used the most for predicting the biomarkers from the transcriptomic data, although the biomarkers found by each of them seemed to vary. Most of the biomarkers found were prognostic in nature and were genes with their upregulation or downregulation having an impact on the proliferation of BC cells. Most of the biomarkers found were oncogenic in nature, ie, their enhanced expression in the bladder tissues aid in the proliferation of BC.

The common limitations that have been encountered in these studies were of the input data; the data collected were old, retrospective in nature, less sample size, or confined to a particular geographical location. So, obtaining data from a large population with different ethnicities can aid in more accurate predictions by ML models. Most of the biomarkers found were not clinically validated and this poses a concern over their clinical usage. Hence, further studies can concentrate on clinically validating these biomarkers for them to be used in the diagnosis of BC. Table 3 denotes the different biomarkers found in these studies, along with their functions, associated pathways, regulators, and roles as biomarkers in other cancers based on the GeneCards database and literature.

**Table 3:** Prominent biomarkers found by ML models using transcriptomics data

Biomaker	Function	Associated pathways*	Regulating transcription factors*	Reported in other cancers	Biomarker type	References
PTHLH	Regulate aspects of cell proliferation, migration and survival	GPCR downstream signalling pathway	AP-1, deltaCREB	Non-small cell lung cancer	Diagnostic and prognostic	[117, 124, 125]
SMAD6	Regulates the TGF-β and BMP signalling pathway	TGF-beta Pathway, BMP receptor signaling pathway	AML1a	Colorectal	Diagnostic	[118, 126, 127]
LDLRAD3	Regulates formation of High endothelial venules	N/A	ATF-2, CUTL1, Evi1, FOXO1, FOXO1a, GATA-1, HNF-1, HNF-1A, IRF-2, Pax-6	Colorectal	Prognostic	[119, 128]
LIG1	Involvement in senescence, DNA replication, cell cycle and p53 pathway	DNA repair pathways	E2F, Elk-1, GATA-2, MAZR MyoD, NF-E2, p45, Nkx2-5 YY1	Breast	Prognostic	[120, 129]
PPP2R2B	Regulates the Wnt pathway	p70S6K Signaling, Beta-Adrenergic Signaling, Wnt signaling pathway	TBP	Breast	Prognostic	[121, 130]
FN1	Functions in regulating apoptosis	Epithelial to mesenchymal transition pathways, ERK Signaling pathway	AP-1, STAT3	Thyroid	Prognostic	[122, 131]
CRYAB	Acts in the process of regulation of cell motility	Cellular heat stress pathway	c-Myc, Max1, NF-kappaB, Sp1	Colorectal	Prognostic	[122, 132]
COL6A1	Converts normal fibroblast cells to cancer-associated fibroblast cells and can activate the TGF-β pathway	Collagen chain Trimerization and Integrin Pathway	AML1a, AP-1, ATF-2, c-Jun, c-Myc, Ik-1, Max1, NF-kappaB, RelA	Osteosarcoma	Prognostic	[122, 133]
FOXP3	Functions as a TF for regulatory T cells and also regulates the HIF-1α gene expression	NF-kappaB Signaling, Wnt/Hedgehog/Notch signalling pathways	AML1a, CREB, deltaCREB	Pancreatic Adenocarcinoma	Prognostic	[123, 134]
TOX	Regulates the tumour microenvironment and is involved in the pathways of immune regulation and T-cell exhaustion	Mesodermal commitment pathway	GATA-1, NF-AT, Pax-4a/5	Lung Adenocarcinoma	Prognostic	[123, 135, 136]

^*^Taken from Genecards database [38]

*N/A* not available

### Proteomics

Proteomes from urinary exosomes are considered excellent sources for bladder cancer biomarkers mainly due to two reasons: one, being a non-invasive detection method, and second, its direct contact with the tumor [137, 138]. However, early studies utilizing proteomic biomarkers for classification were hindered by high intra-tumor heterogeneity and difficulties in comparing biomarkers across diverse studies [138]. This would change as the need for biomarker-based classification, a painless, non-invasive method compared to traditional cystoscopy, is imperative. Traditional urine cytology shows an AUC of only 0.718, coupled with low sensitivity [139]

Most proteomic studies directed to find proteomic biomarkers follow the usual discovery-internal–external validation phases. Efforts have been made to make these phases more accurate and efficient by introducing new screening technologies and, most importantly, using ML models to classify biomarkers that can effectively discriminate. Discovery phases mostly use Capillary electrophoresis coupled to Mass Spectrometry (CE-MS), Sequential Window Acquisition of all Theoretical Mass Spectra (SWATH-MS) by Data-Dependent Acquisition (DDA) or Liquid Chromatography coupled with Tandem Mass Spectrometry (LC–MS/MS) to effectively profile proteins for urine sources. In one study, an aptamer-based screening (called SOMASCAN) of urinary samples was done; This makes it possible for thousands of proteins to be screened at once, improving sensitivity [140]. These proteins, upon identification, usually undergo quantification and sequencing. Validation, yet another important phase, employs ELISA by assessing the fold changes and AUC [141] for internal validation by splitting the dataset into training and testing sets, a well-practiced process. External validation is almost always done on larger cohorts of independent sets through ELISA. In most proteomic studies, many biomarkers are identified as robust.

The advent of Machine learning rises from the need for both higher accuracy and efficiency of classification, as repeated tests are necessary for disease diagnosis and recurrence surveillance [140]. A rather narrow types of algorithms are implemented in classification due to the biomarkers being high dimensional [138], and being a better option compared to unsupervised algorithms. SupporT Vector Machines (SVM) (also Msvm-RFE), with its inherent capability to classify high dimensional parameters, is an excellent resource. Another well-used algorithm is Random Forest Analysis (RFA). Vanarsa, Castillo and Wang et al. constructed an RFA model that rates the importance of the biomarkers that are found, based on their ability to distinguish BC form non-cancer tumors [140]. Principal Component Analysis (PCA) was used to constrict the data to visualize how well the Biomarkers discriminated between different cancer stages, particularly NMIBC, which is usually regarded as an early stage, from MIBC, which refers to the advanced stages.

The models have been shown to have higher levels of AUC and specificity. The study by Frantzi et al, resulted in two biomarker panels for classifying into primary or recurrent bladder cancer. The primary panel, after validation, exhibited an AUC of 0.87 and the Recurrent panel, 0.75, respectively [138]. Interestingly, when incorporating traditional cytology, the AUC of the recurrent panel increased to 0.87, signifying high accuracy. The study also mentioned that biomarkers in both the panels overlap with each other and with previous studies that reported the same set of proteins, further validating the panel. This panel is also great for identifying low-risk recurrent BC biomarkers. Suh et al. noted that a panel of 8 proteins after validation, from DEPs by Data-Independent Acquisition (DIA) methods performed well, with AUC of both their models being 0.845 and 0.842 respectively [141]. Table 4 depicts the key biomarkers found using proteomic studies by ML models, along with their functions, associated pathways, regulators, and their roles as biomarkers in other cancers based on the GeneCards database and literature.

**Table 4:** Prominent biomarkers found by ML models using proteomic data

Biomaker	Function	Associated pathways*	Regulating transcription factors*	Reported in other cancers	Biomarker type	References
Collagen Fragments	Extracellular matrix remodelling, tissue repairing and cell signalling	TGF-β signaling pathway, PI3K/AKT pathway	LEF-1, B-MYB, GLI1	Breast, Pancreas, Gastric, And Colorectal	Diagnostic	[138, 142, 143]
Apolipoprotein A-I	Liquid transport, anti-inflammation, (potential) role in tumor cell proliferation and angiogenesis	Plasma lipoprotein assembly, remodeling, and clearance and Response to elevated platelet cytosolic Ca2 +	C/EBPalpha, COUP HNF-4alpha1/2, PPAR-gamma1/2	Ovarian, Breast, Gallbladder, Liver, Colon, Cervix, Uterus, Prostrate, Kidney, Lung, Head & Neck	Diagnostic and recurrence	[138, 140, 144, 145]
Hemoglobin chains	Oxygen transport, (potential) indicator of hematuria	ERK1/2, P38/MAPK, and JNK pathways	C/EBPbeta, FOXD1/3b, GATA1, IRF1, STAT3, TBP, USF1	Lung, Breast,	Primary and recurrent. More prominent in primary	[138, 146]
Fibrinogen A (FGA)	Blood clotting, indicator of disrupted coagulation pathways	RAS, FAK–ERK, integrin-AKT, and TLR7/8 pathway	E47, Evi-1, FOXO3, ISGF-3, Pbx1a, STAT3, Tal-1	Lung, Gastric, Colorectal	Diagnostic	[141, 144, 147]
Matrix Metalloproteinases (MMPs)	ECM degradation, associated with disease progression and aggressiveness	EGF receptor pathway	AP-1, ATF-2, c-Jun CREB, SRF	Breast, Pancreas, Lung, Bladder, Colorectal, Ovarian, Prostrate, Brain	Diagnostic and Prognostic	[138, 140, 144, 148]
α-2-macroglobulin (A2M)	protease inhibition and transport of cytokines	Classical pathway of the complement system	AREB6, CREB, FAC1, GR, Lmo2, STAT5A	Leukemia, Prostrate	Diagnosis and recurrence	[141, 144, 149]
Uromodulin	function not fully understood, but it may play a role in preventing kidney stones and urinary tract infections	Metabolism of proteins and transport to the Golgi and subsequent modification	AhR, Brachyury, HFH-1, Nkx3-1, XBP-1	Prostrate	Muscle invasive (advanced) vs Non-Muscle invasive (early)	[150, 151]
Inter-Alpha-Trypsin Inhibitor Heavy Chain 2 (ITIH2)	Regulate inflammation and extracellular matrix remodeling	PI3K/AKT signaling pathway, Regulation of Insulin-like Growth Factor (IGF) transport and uptake by Insulin-like Growth Factor Binding Proteins (IGFBPs) and Metabolism of proteins	FOXJ2, HFH-3, MyoD, MZF-1, Olf-1, PPAR-gamma1, RSRFC4 STAT5A	Glioma, Ovarian, Liver	Diagnostic	[143, 144, 152]
Complement components	Inflammation and defense against pathogens. Dysregulation has been implicated in cancer development and progression	classical and complement pathway	AP-1, ATF-2, c-Jun PPAR-gamma1/2, STAT3	Lung, Colorectal, Ovarian, Gastric, Oropharyngeal	Diagnosis and recurrence	[138, 143, 144, 153]
Fascin-1 (FSCN1)	Embryogenesis, tissue repair, fibrogenesis, cell motility invasion, and immune response factor	Cytokine signalling, innate immune system	CREB, p53	Breast, Ovarian, Pancreatic, Skin, Lung, Gastric, Esophageal, Kidney, Colon And Prostrate	Recurrence	[138, 144, 154]

^*^Taken from Genecards database [61]

As much as the identification of proteomic biomarkers is of great importance for efficiency and diagnostic milestones for urothelial cancer therapy, several limitations and shortcomings loom. The most important one is the limited transition to clinical settings. These identified biomarkers are not yet commonly employed in clinical practice for diagnosis, staging, and recurrence monitoring. More focus should be placed on clinical trials and longitudinal studies to validate these biomarkers in real-world scenarios [155]. This can bridge the gap between research findings and clinical application. The narrow set of ML algorithms being used, mainly just SVM and Random Forest, albeit reliable, can have limitations in handling very complex data structures. The high amount of false-positives in recurrent detection remains another problem [144]. Balancing specificity and sensitivity are critical factors for improved models. Additionally, scarce amounts of biomarkers identified for early-stage cancer is another concern. Many biomarkers have been identified for diagnosing and monitoring advanced urothelial carcinoma, however, very few studies focus on early-stage prediction of urothelial carcinoma, particularly NMIBC.

### Metabolomics

Metabolomics, the study of small molecules involved in biological processes, is another great approach for bladder cancer diagnosis, staging, and recurrence prediction, using biomarkers, here called ‘metabolomes’. Urine and serum are commonly used biofluids for metabolomic profiling as they contain metabolites that are reflective of changes in bladder tissue.

Several studies have used various analytical platforms, including Ultra-performance liquid chromatography time-of-flight mass spectrometry (UPLC-TOF–MS) and Gas chromatography-mass spectrometry (GC–MS), to analyze urine metabolites and to differentiate bladder cancer from controls. Shao et.al, used UPLC-TOF–MS and a 4-step marker discovery pipeline to identify 6 potential bladder cancer markers. This pipeline includes selection by detection count, three-parameter Gaussian fitting, statistical test, and AUC. Building a decision tree model based on these 6 markers, the model achieved an accuracy of 76.60% and specificity of 86.67% [156].

Another study by Wang.et.al, utilized UPLC-MS to profile urine metabolites from bladder cancer patients and healthy controls. Through Orthogonal Projections to Latent Structures Discriminant Analysis (OPLS-DA) analysis and univariate analysis, 19 metabolites were identified as potential biomarkers. Further analysis done using SVM, Random Forest and Boruta algorithms narrowed down the metabolites to 11. A logistic regression model with these 11 biomarkers showed an AUC of 0.983 and a specificity of 100%, demonstrating exceptional diagnostic capability [157].

GUDCA and Palmitoylcarnitine are two of the most prominent biomarkers among metabolomes. GUDCA is a bile acid that has roles in fat digestion and absorption, solubilizing cholesterol, and facilitating its elimination from the body. It is found to be significantly upregulated in bladder cancer patients [157], perhaps indicating altered bile acid metabolism or a response to cellular stress induced by the tumor. Palmitoylcarnitine is involved in the transport of fatty acids into mitochondria. It is also observed to be highly upregulated in cells (almost tenfold) in bladder cancer patients [157, 158], possibly from the Warburg effect.

Serum Metabolomics, although less common, also holds great promise in diagnosis. Troisi et al. constructed ensemble ML models using serum metabolomics profiles to differentiate between cancer patients and controls, lo-grade (G1-2) and high-grade (G3) cancers, and different degrees of muscle invasiveness. These ensemble models demonstrated an accuracy of at least 80% across classification tasks. The study also identified Waskey metabolic pathways contributing to bladder cancer, including perturbations in amino acid, glutathione, glyoxylate, and dicarboxylase metabolism [159].

Even with astounding progress in using metabolomic biomarkers for diagnosis, challenges still loom. Variability in study design, analytical platforms, and data analysis methods across different studies makes it quite difficult to compare and validate results. Using larger cohorts that are well-defined, implementing standardized protocols for sample collection, preparation, and analysis, or focusing on validating identified biomarker panels through independent cohorts and clinical trials can hopefully facilitate the translation of metabolomic biomarker panels into clinical tools for bladder cancer diagnosis, staging, and recurrence. Table 5 contains the different biomarkers found in these studies, along with their functions, associated pathways and their roles as biomarkers in other cancers based on the GeneCards database and literature.

**Table 5:** Prominent biomarkers found by ML models using metabolomic data

Biomarker	Function	Associated pathways*	Reported in other cancers	Biomarker type	References
ADENOSINE MONOPHOSPHATE (AMP)	Activates AMPK, which may give tumor cells a growth advantage by controlling the metabolic adaptability of cells	ATP and adenosine signaling pathways	N/A	Diagnosis	[157]
GLYCOURSODEOXYCHOLIC ACID (GUDCA)	Bile acid, overexpression in urine, leads to worse bladder conditions	Cellular proliferation**,** mTOR/S6K1 pathway	Colon	Diagnosis	[157, 160]
CHENODEOXYCHOLIC ACID (CDCA)	Bile acid, overexpression in urine leads to worse bladder conditions and promotes epithelial-mesenchymal transition	Lipid peroxidation, ROS/p38 MAPK/DGAT1 pathway	Acute Myeloid Leukemia	Diagnosis	[157, 161]
GLYCOCHENODEOXYCHOLIC ACID (GCDCA)	Bile acid, overexpression in urine-leads to worse bladder conditions	Cellular proliferation	Hepatocellular	Diagnosis	[157, 162]
Palmitoylcarnitine	Induces inflammatory cytokines, drives bladder tumor development	Metabolism of fatty acids, Ca^2+^ influx	Prostrate	Diagnosis	[157, 163]
Imidazoleactic acid	Derived from oxidizing histamine, which is released by mast cells during inflammation	Histidine metabolism	Rectal	Diagnosis	[156, 164]
Taurine	Involved in osmoregulation, antioxidant defence and cell signaling	Alanine-3-sulfinate pathway, NF-κB pathway	Lung, Breast	Diagnosis	[156, 165, 166]
Histidine	Derives histamine and therefore imidazolic acid, which was found enriched in bladder cancer metabolomes	Cell signaling, Protein synthesis	Prostrate, Ovarian	Diagnosis	[156, 167]
Lactate	High levels associated with Warburg effect in cancer cells	PD-1/PD-L1 pathway, cellular metabolism	Esophageal, Colorectal, Hepatocellular, Pancreatic, Gastric, Breast, Prostrate, Ovarian, Glioma	Diagnosis	[159, 168]
Glucose	Primary energy source	Glycolysis, TCA	Hepatocellular, Pancreatic, Ovarian, Breast, And Colorectal	Diagnosis	[159, 169]

^*^Taken from Genecards database [61]

*N/A* not available

## Multi-omics data integration in bladder cancer research

Attaining a comprehensive understanding of the intricate biological processes in cancer requires the integration of variations across multiple levels, including genomics, epigenomics, transcriptomics, metabolomics, and proteomics. This synergy provides a detailed catalogue of these processes, enabling classification, disease subtyping, prediction of biomarkers, and obtaining insights into the pathophysiology of the disease more evident [170]. For instance, identifying an SNP associated with the disease outcome, Copy number variations and its influence on gene expression, epigenetic markers, proteins, and metabolites all give valuable diagnostic and prognostic markers [171]. These synergic effects can be observed, identified, and interlinked by utilizing novel AI and machine learning algorithms. For instance, MOGONET is a supervised multi-omics integration algorithm to analyze and classify data. It can learn omic-specifics using graph convolutional networks (GCN) and efficiently perform cross-omics correlations using view correlation discovery network (VCDN) [172]. Furthermore, Zang et al. introduced a novel approach by combining machine learning and deep learning for multi-omics analysis of MIBC to enhance the prognosis of bladder cancer. Autoencoders were used to integrate gene expression, CNVs, miRNA expression, and DNA methylation from TCGA-MIBC data. Additionally, Machine learning algorithms, including random forest, Naïve Bayes, k-NN, and Adaboost, classified patients into high-risk and low-risk subtypes. Significant genomic, immune component, and pathway differences were observed between groups, and importantly, KRT7 was identified as a key biomarker for MIBC from the RNA and protein levels [173].

Moreover, the multi-omics integration methods can be categorized into two types: horizontal and vertical. Horizontal integration is studying the same omics (e.g., transcriptomics) across different sample groups, allowing comparative analysis and identification of condition-specific variations. In contrast, vertical integration is used for multi-omics analysis, where different omics layers (e.g., genomics, proteomics, transcriptomics) are integrated for the same set of samples but contain different features [174]. The vertical integration methods can be classified into network, fusion, correlation-based, and similarity-based methods. Several computational tools have been developed to facilitate multi-omics integration. iClusterPlus is a statistical tool used to systematically cluster multi-omics data and identify shared patterns, such as identifying tumor subtypes. MIXOmixs, an R package that enables integration and analysis of multi-omics data. MoFA (Multi-omics factor analysis) employs Bayesian tool used to integrate multi-omics data, focusing on factor models while Multiple factor analysis (MFA) integrates omics datasets by projecting them in a low-dimensional space. Similarity network fusion (SNF), which uses a network approach to first create individual networks and later using a non-linear network fusion approach, fuses them into a single similarity network [175].

Several BC studies have successfully integrated multi-omics data to identify key biomarkers, classify tumor subtypes, and improve prognostic predictions. A study conducted by Chu et al. [176] on muscle-invasive urothelial cancer utilized 10 clustering algorithms, including SNF, COCA, IntNMF, PINSPlus, iClusterBayes, NEMO, moCluster, and LRA to integrate genomics, transcriptomics, and epigenomics datasets. The candidate genes obtained through these approaches were further analyzed using prognostic ML models for patient prognosis and screening potential therapeutic targets [176]. Similarly, another study with MIBC with mutation data, DNA copy number, RNA-Seq, and methylation data were analyzed with the iClusterBayes method. With the integrative analysis, it was found that patients with low expression of *MTAP/CDKN2A/2B* had a low response rate to immunotherapy and worse survival, revealing intrinsic MIBC subtypes and prognostic biomarkers [177]. Another study conducted by Shi et al [178], with BC employing whole exome sequencing, RNA sequencing, reduced-representation bisulfite sequencing (RRBS), and oxidative reduced-representation bisulfite sequencing (oxRRBS). The integrative analysis depicted the increased involvement of epigenetic alterations compared to genetic mutations. Similarly, Transcriptomic, proteomic, and protein acetylation sequences were integrated to identify key prognostic genes in BC. A risk model and nomogram incorporating clinical factors were developed, aiding in patient stratification and highlighting the potential of multi-omics and ML in prognosis and therapeutic decision-making [178].

## Challenges and research directions

While machine learning (ML) has significantly enhanced biomarker identification by improving efficiency, accuracy, and scalability, several challenges remain. Overfitting is one of the most common. Multi-omics datasets, despite being highly dimensional, are often limited to less sample size, leading to the same. In such situations, it is prudent to implement k-fold cross-validation (fivefold or tenfold) during model training to evaluate robustness. For regularization, LASSO (L1 regularization) and Ridge (L2 regularization) are used in penalizing over-complex models [179]. Further, the other major hurdle is the lack of interpretability in ML models, particularly black-box models like deep learning and gradient boosting, which can hinder clinical adoptions. A promising solution involves explainable AI methods such as Shapley Additive exPlantations (SHAP) and Local interpretable Model-agnostic explanation (LIME) that can bridge the interpretability gap. For example, applying SHAP to proteomic ML models revealed that APOE and CD44 drive predictions by regulating EMT pathways, allowing biologically credible validation [180, 181]. Additionally, reproducibility and data quality continue to be a tough hurdle for robust ML models. A 2022 study demonstrated that only 22 of the 74 AI-derived biomarkers in bladder cancer studies were validated across independent cohorts [182]. ML models often overlook pre-analytical variables (such as urine sample handling) and platform-specific biases (eg. SWATH-MS vs. LC–MS/MS) [183]. Similarly, the barrier to clinical integration remains, with less than 5% of ML models being integrated into clinical workflows due to interoperability challenges with electronic health records and regulatory hurdles [184]. To improve generalizability, advanced architectures such as Deep MOIS-MC have been developed to enhance cross-omics correlation, identifying consensus biomarkers across genomics, proteomics, and metabolomics [185]. Additionally, deploying ML models as an interoperable docker container within hospital systems can streamline adoption. A pilot study integrating the DeepCysto AI tool into cystoscopy suites reduced false-positive rates by 22% through real-time contour analysis [180].

Most importantly, the biomarkers obtained from ML applications have to undergo clinical validation to ensure clinical translation. Initially, in the discovery phase, potential biomarkers are identified using ML-based analysis of genomic, proteomic, or metabolomic datasets. Following this, analytical validation is conducted to assess the precision, reproducibility, and specificity of biomarkers in laboratory settings using techniques like Immunohistochemistry, Western Blotting, Quantitative Polymerase Chain Reaction (qPCR), RT-PCR, Enzyme-Linked Immunoassay (ELISA), or Florescence In Situ Hybridization (FISH). Subsequently, clinical validation establishes the biomarker’s correlation with clinical outcomes and disease phenotypes. Biomarkers then undergo clinical utility assessment, evaluating their impact on patient outcomes, diagnosis, or prognosis. Lastly, regulatory bodies such as the Food and Drug Administration (FDA) or the European Medicines Agency (EMA) approve these biomarkers with adequate evidence. However, challenges exist, including non-standardized isolation techniques and lack of reproducibility across studies [186, 187]. These challenges can be overcome by establishing standardized protocols, consistency in sample processing, and enhanced data interpretation, and the patient data should be encrypted. Additionally, conducting multi-center studies with diverse cohorts and optimizing workflows will enhance reproducibility and clinical applicability. Future advancements, including deep learning for unveiling complex biological interactions and integration of real-time multi-omics profiling with ML models, may enable dynamic monitoring of treatment response through biopsy platforms, hopefully replacing surveillance cystoscopies within the near future.

## Conclusion

Bladder cancer poses a significant health burden, particularly in developed countries, which highlights the urgent need for improved diagnostic and prognostic strategies. Utilizing ML algorithms on the multi-omics data, including genomics, epigenomics, transcriptomics, proteomics, and metabolomics, offers a wide range of applications in biomarker identification. ML enables the identification of novel biomarkers that enhance early detection, define risk stratification, predict recurrence, classify cancer subtype, and improve prognostic accuracy. However, challenges such as data integration complexities, model interpretability, and clinical validation must be addressed to ensure the reliability and applicability of these approaches. Future models need to leverage learning frameworks to harmonize datasets while preserving patient privacy, addressing critical gaps in ethnic/geographic diversity that currently limit biomarker generalizability. Advances in explainable AI tools like SHAP and LIME will bridge the interpretability gap, enabling more clinicians to trust ML-derived biomarkers. As AI-driven methodologies continue to evolve, their seamless integration into clinical workflows could significantly improve patient outcomes, reduce reliance on invasive diagnostics, and advance the era of precision oncology in bladder cancer.

## Data Availability

No datasets were generated or analysed during the current study.
